# eIF2 interactions with initiator tRNA and eIF2B are regulated by post-translational modifications and conformational dynamics

**DOI:** 10.1038/celldisc.2015.20

**Published:** 2015-08-11

**Authors:** Victoria Beilsten-Edmands, Yuliya Gordiyenko, Jocky CK Kung, Shabaz Mohammed, Carla Schmidt, Carol V Robinson

**Affiliations:** 1 Department of Chemistry, University of Oxford, Oxford, UK; 2 Department of Biochemistry, University of Oxford, Oxford, UK

**Keywords:** acetylation, eukaryotic translation initiation, mass spectrometry, phosphorylation

## Abstract

Translation of messenger RNA (mRNA) into proteins is key to eukaryotic gene expression and begins when initiation factor-2 (eIF2) delivers methionyl initiator tRNA (Met-tRNA_i_^Met^) to ribosomes. This first step is controlled by eIF2B mediating guanine nucleotide exchange on eIF2. We isolated eIF2 from yeast and used mass spectrometry to study the intact complex, and found that eIF2β is the most labile of the three subunits (eIF2α/β/γ). We then compared conformational dynamics of the ternary complex eIF2:GTP:Met-tRNA_i_^Met^ with apo eIF2 using comparative chemical cross-linking. Results revealed high conformational dynamics for eIF2α in apo eIF2 while in the ternary complex all three subunits are constrained. Novel post-translational modifications identified here in both eIF2 and eIF2B were combined with established sites, and located within protein sequences and homology models. We found clustering at subunit interfaces and highly phosphorylated unstructured regions, at the N-terminus of eIF2β, and also between the eIF2Bε core and catalytic domains. We propose that modifications of these unstructured regions have a key role in regulating interactions between eIF2 and eIF2B, as well as other eIFs.

## Introduction

Translation initiation in eukaryotes is a highly regulated process and requires the interplay of several translation initiation factors [1, 2]. It begins with delivery of Met-tRNA_i_^Met^ to the ribosome by eukaryotic initiation factor 2 (eIF2) after formation of the ternary complex eIF2:GTP:Met-tRNA_i_^Met^. Met-tRNA_i_^Met^, accompanied by other initiation factors, is brought to the P-site of the 40S ribosomal subunit and is followed by binding of the mRNA 5′-untranslated regions and scanning for an AUG start codon [3]. Base-pairing of the Met-tRNA_i_^Met^ anticodon with the AUG start codon of the mRNA triggers hydrolysis of eIF2-bound GTP followed by dissociation of eIF2-GDP together with other initiation factors. After joining of the 60S subunit, the ribosome is transferred to the elongation phase of protein translation [4]. Nucleotide exchange on eIF2 is then performed by interactions with eIF2B, the nucleotide exchange factor, preparing eIF2 for a new round of translation initiation.

The majority of the eIFs consist of multisubunit protein complexes with defined protein topology. eIF2 is highly conserved and composed of three subunits (α, β and γ) encoded by *SUI2*, *SUI3* and *GCD11* in yeast. There is no high-resolution structure for intact yeast eIF2 available, although structural insights have been obtained from an X-ray structure of the archaeal homologue (aIF2) from *Sulfolobus solfataricus*[5], and it is very likely that eukaryotic eIF2 assembles in a similar manner. In this structure, IF2 adopts an elongated form without contact between the α- and β-subunits. The core of this complex is formed by the γ-subunit bound to domain 3 of the α-subunit and the N-terminal helix of the β-subunit [5]. Formation of the ternary complex is mediated by the γ-subunit, which binds GTP and Met-tRNA_i_^Met^ directly [6], whereas the α- and β-subunits stabilise Met-tRNA_i_^Met^ interactions [7].

eIF2B is much larger than eIF2 and contains five protein subunits denoted α, β, γ, δ and ε (encoded by *GCN3*, *GCD7*, *GCD1*, *GCD2* and *GCD6* in yeast). eIF2B forms a decameric complex containing two copies of each subunit in human [8] and in yeast [9]. Guanine nucleotide exchange function is assigned to ε- and γ-subunits [9–11], whereas α-, β- and δ-subunits form an additional regulatory subcomplex [12]. In the absence of full-length high-resolution structures of the eIF2B subunits, valuable insights have been obtained from homologous structures [13], allowing generation of simplified models [9].

While translation initiation is known to be regulated by interactions between the various eIFs, other factors such as post-translational modifications (PTMs) have been reported, although their roles in these interactions are not fully understood. Although inhibition of nucleotide exchange on eIF2 has been studied extensively and assigned to phosphorylation of the α-subunit [11, 12, 14], additional regulatory mechanisms initiated by changes in PTMs have not been deduced. Many of the PTMs reported to date have originated from large-scale proteomic studies of entire organisms or cells [15–18]. A recent quantitative study revealed the phosphorylation status of selected eIFs purified from HeLa cell lysates harvested at the log phase and indicated the phosphorylation sites that are important for translation initiation [19]. A comprehensive study of acetylation and phosphorylation sites in yeast eIF2 and eIF2B and the relative locations of these sites on interacting subunits have not been reported to date.

Because of its central role in translation initiation, eIF2 represents an important control point for protein synthesis during adverse conditions. Phosphorylation of Ser51 on the α-subunit [20] is key to this control and effects tight binding of eIF2 and eIF2B, thus preventing initiation of a new round of translation [12, 21]. Recently, we studied the architecture of the eIF2–eIF2B complex and showed that interactions between these two initiation factors involve elongated eIF2 bringing the GTP binding site of the γ-subunit in close proximity to the catalytic domain of eIF2Bε, thus providing a platform for nucleotide exchange [9].

Here we extend our investigation to this checkpoint for protein translation by investigating eIF2, formation of the ternary complex and the location of PTMs in both eIF2 and its nucleotide exchange factor eIF2B. We combined mass spectrometry (MS) and comparative chemical cross-linking to gain insights into protein interactions within apo eIF2 and the ternary complex. We found that apo eIF2 is highly flexible in solution with the β-subunit being only loosely associated. After binding Met-tRNA_i_^Met^, the ternary complex adopts a rigid structure alleviating flexibility of the protein subunits. Placing PTMs identified here, together with others reported previously, in a structural context reveals clustering of PTMs and regions of extensive phosphorylation, primarily in flexible loops, suggesting regulatory roles in mediating interactions in translation initiation. Overall, our results allow us to generate an eIF2 model that adopts both flexible and rigid conformations, and is fine-tuned by its phosphorylation status either to execute distinct functions or to interact with the nucleotide exchange factor eIF2B or other eIFs.

## Results

### MS of trimeric eIF2 reveals weak association of the β-subunit

**Figure 1:**
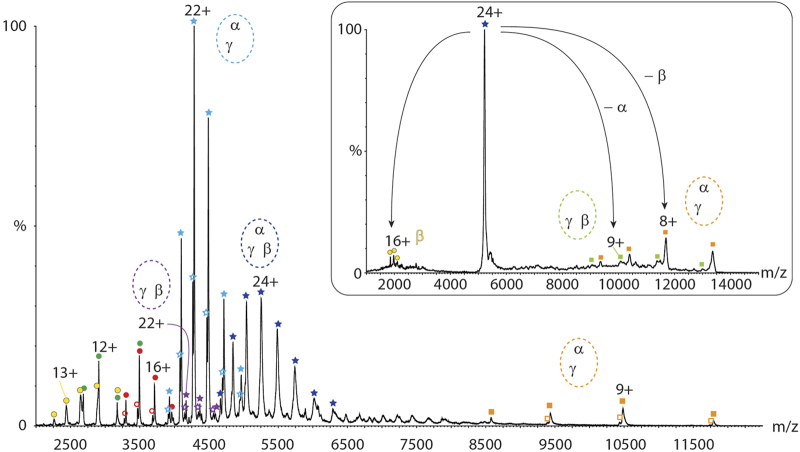
Mass spectrum of trimeric eukaryotic initiation factor 2 (eIF2). His-tagged eIF2 was purified from yeast. The mass spectrum shows intact trimeric eIF2 (dark blue stars), α/γ and β/γ dimers (light-blue and purple stars), protein subunits (α, green; γ, red; and β, yellow) and stripped α/γ dimer (orange). The dimeric species and γ-subunit show two populations corresponding to bound (filled) and unbound (unfilled) GDP (average Δm 461 Da). The insert shows a tandem mass spectrum of intact eIF2 (charge state 24+). Two stripped complexes were observed, the predominant α/γ dimer (orange) and the low-abundant β/γ dimer (light-green). Highly charged β-subunit (yellow) was also observed.

We purified intact eIF2 from a yeast *Saccharomyces cerevisiae* strain engineered to overexpress the three protein subunits including a His-tag on the γ-subunit. After utilising a His-tag strategy (see Materials and Methods section), SDS-polyacrylamide gel electrophoresis and liquid chromatography-coupled tandem-MS (LC-MS/MS) confirmed the presence of all proteins with high confidence (Supplementary Figure S1 and Supplementary Table S1). We next used a mass spectrometer modified for transmission of high-mass protein complexes [22] to study intact eIF2 (Figure 1). The mass spectrum shows the presence of all three protein subunits, two dimeric species as well as a peak series corresponding to intact eIF2 in 1:1:1 stoichiometry. The γ-subunit, and all subcomplexes containing this subunit, showed peak splitting assigned to two populations. The mass difference between these peaks series corresponds to bound and unbound GDP present in the purification buffer relating to the GTP/GDP binding site in the γ-subunit. The masses of the protein subunits and complexes obtained agree well with their theoretical masses (Supplementary Table S2) and confirm that intact eIF2 is trimeric with one copy of each protein subunit, which is in agreement with our previous data [9].

Evaluating the presence of subcomplexes and their relative intensities demonstrates that the β-subunit dissociates readily from the intact complex. The α/γ dimer formed in solution shows a high intensity, whereas its counterpart, the β/γ dimer, is of very low abundance. This observation is further strengthened by the presence of the stripped α/γ dimer formed in the gas phase under these mild activation conditions (Figure 1). Selecting the 24+ charge state of the intact eIF2 complex for dissociation in the collision cell yields primarily the stripped α/γ dimer and highly charged β-subunit (Figure 1, insert). In contrast to the β-subunit, the α-subunit is not readily dissociated from the trimeric complex, thereby implying more stable subunit interactions between α/γ than β/γ.

### Chemical cross-linking and homology modelling

To study protein interactions in eIF2, we subjected the intact complex to chemical cross-linking. We used the lysine-specific cross-linker bis(sulfosuccinimidyl)suberate (BS3) in its deuterated (d4) and non-deuterated (d0) form to obtain specific peak pairs for cross-linked peptides in the MS spectra and thus to facilitate data analysis. The eIF2 complex was cross-linked with a 1:1 mixture of BS3-d0/d4. The proteins were subsequently hydrolysed with trypsin and the mixture of peptides and cross-linked peptides was analysed by LC-MS/MS. After database searching, we identified 291 potential cross-linked peptides, and manual inspection of MS and MS/MS spectra revealed 43 false positives, giving us a false discovery rate of 14.78% for this experiment (Supplementary Table S3).

To view the intra- and inter-protein interactions in a structural context, we made use of homology models that were based on models we reported previously [9]. These models were obtained from SWISS-MODEL and Modeller web servers using the crystal structures of archaeal aIF2 from *Sulfolobus solfataricus* (α-subunit; PDB ID 3CW2 [23] and β-subunit; PDB ID 2QMU [5]) and *Pyrococcus abyssi* (γ-subunit; PDB ID 1KK1 [24]) as templates (Supplementary Table S4). Although these homology models do not represent the full protein sequences, the interaction interfaces and structured regions of these models give insights into the structures of eIF2 protein subunits from yeast.

**Figure 2:**
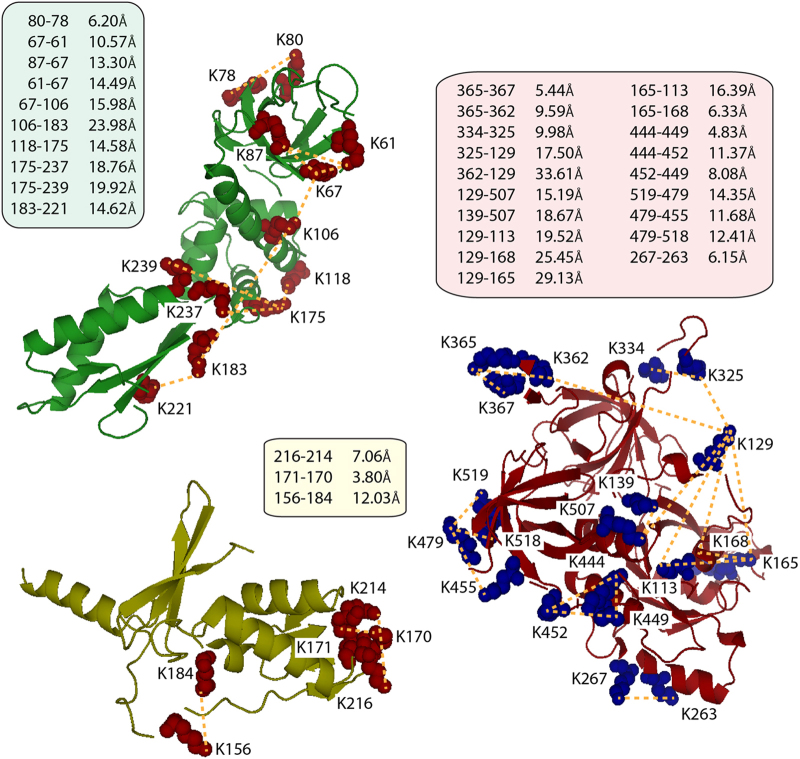
Homology models of eukaryotic initiation factor 2 (eIF2) protein subunits. Homology models for α- (green), β- (yellow) and γ-subunits (red) are shown. Intra-protein cross-links are shown as dotted lines and cross-linked lysine residues are shown as space fillings (α and β, red; and γ, blue). Cα-distances in Å are given for the projected cross-links.

Of the 47 intra-protein cross-links obtained (Supplementary Table S3), 32 cross-links could be projected onto the homology models of α-, β- and γ-subunits (Figure 2). The remaining 15 intra-protein cross-links are either located in protein regions, which are not included in the homology models (Supplementary Table S4), or their peptide sequences were not unique and could not be assigned unambiguously to a certain amino acid residue in the protein sequence (Supplementary Table S3). To verify that these cross-links are in a reasonable range, we measured the Cα-distances between the cross-linked lysine residues. The BS3 cross-linker has a length of 11.4 Å, adding to this 6.5 Å for each cross-linked lysine side chain (≈24.4 Å) and allowing for conformational dynamics of the proteins yields a maximum distance of ~35 Å. The majority of the intra-protein cross-links are well below this threshold, only three cross-links in the γ-subunit are close to this upper limit (Figure 2). These cross-links (K129:K165, K129:K168 and K129:K362) are located in flexible loops (K129 and K362), and conformational dynamics within the protein likely account for this increased cross-linked distance. The intra-protein cross-links that we obtained suggest their appropriate implementation to study protein interactions in the eIF2 complex and confirm our homology models.

### eIF2 is flexible in solution

**Figure 3:**
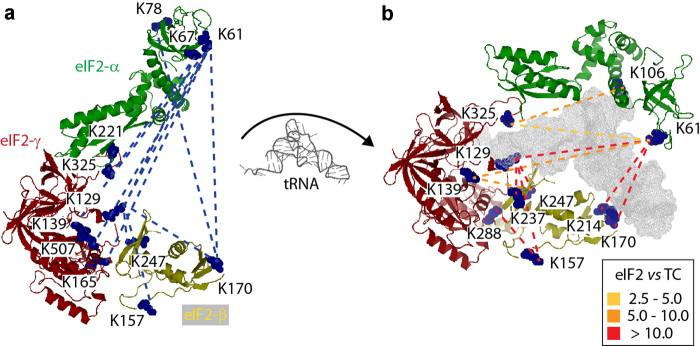
Cross-linking of eukaryotic initiation factor 2 (eIF2) and formation of the ternary complex. (**a**) The homology models of α-, β- and γ-subunits were aligned with the archaeal homologue of eIF2 (PDB ID 3CW2). Cross-linking apo eIF2 reveals several interactions between the α-subunit and β- and γ-subunits, showing that eIF2 is highly flexible in solution. (**b**) Homology models of α-, β- and γ-subunits were aligned with the archaeal homologue of the ternary complex (TC; PDB ID 3V11). Comparative cross-linking of apo eIF2 versus the TC reveals multiple inter-subunit cross-links with reduced intensities after binding Met-tRNA_i_^Met^. Changes in cross-linking intensities are colour coded; values 2.5–10 represent cross-linking intensities that are higher in eIF2 compared with the TC.

In addition to intra-protein cross-links, we also gained insights into protein interactions between the three protein subunits. To visualise these interactions, we used the homology models of α-, β- and γ-subunits and aligned them to the archaeal structural homologue aIF2 (PDB ID 3CW2; Figure 3a). The model shows eIF2 in an elongated conformation with eIF2γ in the centre of the complex and the α- and β-subunits forming a pocket for Met-tRNA_i_^Met^ binding.

Twelve of our 26 inter-subunit cross-links could be projected onto this eIF2 model and are consistent with the α-subunit making multiple interactions with the β- and γ-subunits. These multiple interactions revealed by our cross-linking imply that eIF2 is highly dynamic in solution (Figure 3a). This flexibility is presumably induced by domains 1 and 2 of the α-subunit, which are linked to the flexible domain 3 [25]. Our cross-linking results are therefore complementary to our mass spectra obtained from intact eIF2, which showed that the β-subunit readily dissociates from the trimeric complex, indicating its weak association with the α/γ complex.

### Formation of the ternary complex

Having established the structural properties of apo eIF2, we studied interactions of eIF2 in the context of the ternary complex, that is, bound to Met-tRNA_i_^Met^ and GTP. For this, the tRNA_i_^Met^ was charged with methionine (see Materials and Methods section) and incubated in excess with purified eIF2 complex in the presence of GDPNP, a non-hydrolysable GTP analogue, to assemble the ternary complex. We then used chemical cross-linking to explore changes in protein interactions upon Met-tRNA_i_^Met^ binding to the trimeric eIF2 complex.

We used the same cross-linking workflow as described above. After manual validation, we identified 43 cross-links (Supplementary Table S5) with an false discovery rate of 22.1%. We aligned the homology models of the eIF2 protein subunits with the archaeal homologue of the ternary complex (PDB ID 3V11; Supplementary Figure S2). We were able to project 34 cross-links onto this model. Interestingly, however, only two of these were inter-protein cross-links. Most of the cross-links are located on the exposed surface of the ternary complex, on the opposite face to the Met-tRNA_i_^Met^ binding cleft, and do not reveal protein interactions within the ternary complex.

### Comparative cross-linking reveals a rigid structure of the ternary complex

As we were unable to address conformational changes in eIF2 that occur upon Met-tRNA_i_^Met^-binding, we used a comparative cross-linking strategy [26], which involves a quantitative comparison of protein interactions in the two complexes (apo eIF2 versus the ternary complex; Supplementary Figure S3). We cross-linked apo eIF2 with BS3-d0 and the ternary complex with BS3-d4, and pooled aliquots of the two cross-linked complexes in a 1:1 ratio. After separation on a one-dimensional gel, the proteins were subjected to tryptic digestion and the resulting cross-linked peptides were identified following LC-MS/MS (see Materials and Methods section). We are therefore able to compare directly the intensities of light and heavy cross-linked peptides from eIF2 and the ternary complex. By generating extracted ion chromatograms and comparing the ratios of the peak areas (d0:d4), we obtained quantitative values for changes in cross-linked peptides.

We identified 79 cross-linked peptide pairs of which 60 showed a >2.5-fold change in intensity (Supplementary Table S6 and Supplementary Figure S4). Significantly, the number of inter-subunit cross-linked peptides assigned in this comparative cross-linking experiment is much higher than for the ternary complex alone (21 compared with only 2 assigned for the cross-linked ternary complex, see above). This difference arises as following Met-tRNA_i_^Met^ binding peak intensities of the inter-subunit cross-linked peptides were significantly reduced compared with apo eIF2. The presence of cross-linked peptides derived from apo eIF2 in the same spectrum as those from the ternary complex that enabled us to assign d4 cross-linked peptides derived from the ternary complex, although their low intensity would preclude assignment in the absence of d0 peptide. We found that 54 cross-links within the Met-tRNA_i_^Met^-binding pocket were substantially reduced showing that these sites are no longer accessible to the cross-linking reagent. Accordingly, the intensities of some of the cross-linked peptides on the outside of the ternary complex were increased relative to the apo form. These increased intensities were mainly observed in the α-subunit and arise due to the more rigid structure in the ternary complex stabilising residues for cross-linking (Supplementary Figure S4). Together, these results show that Met-tRNA_i_^Met^-binding affects eIF2 flexibility, restricts access to residues in the Met-tRNA_i_^Met^ binding site and that the ternary complex adopts a more rigid structure than apo eIF2, presumably to hold Met-tRNA_i_^Met^ in place for transfer to the 40S ribosome (Figure 3b).

### PTMs of eIF2 and eIF2B

Met-tRNA_i_^Met^ binding to eIF2 and its release from the ternary complex as well as recycling of eIF2 by eIF2B represent important checkpoints during translation initiation in eukaryotes. Having established the structural features of eIF2 and the ternary complex, we set out to search for novel regulatory mechanisms in this system. Phosphorylation is probably the most characterised PTM in eukaryotes and also has an important role in regulation of eIF2 by inhibiting nucleotide exchange by eIF2B [21]. To date, however, only phosphorylation of Ser51 in eIF2α has been described in eIF2 regulation. We therefore studied phosphorylation in both eIF2 and eIF2B complexes to identify potential regulatory sites.

First, we used titanium dioxide (TiO_2_) enrichment of phosphopeptides followed by LC-MS/MS analysis (see Materials and Methods section). After database searching, potential phosphorylated peptides were manually verified by inspection of their MS/MS spectra. We identified 10 and 7 phosphorylation sites in eIF2 and eIF2B, respectively. We also used a high-sensitivity, high-resolution mass spectrometer without phosphopeptide enrichment. In this way, we identified eight phosphosites in eIF2 and eIF2B directly from the peptide mixtures. Three of these sites were not identified after TiO_2_ enrichment allowing us to extend our list of phosphosites (Table 1). Following these two strategies, we identified 20 phosphosites in total, 13 sites in eIF2 and 7 in eIF2B subunits. Most of the phosphorylated residues identified are serines (17 sites, 85%), whereas threonine and tyrosine residues are phosphorylated to a much lower extent (two and one, respectively).

A crosstalk between phosphorylation and acetylation has been described in eukaryotes and bacteria (for review, see Beltrao *et al*. [27] and Soufi *et al.* [28]), We therefore also studied acetylation in these eIFs. We identified and verified nine acetylation sites in eIF2 and eIF2B subunits directly from the peptide mixtures when using a high-sensitivity, high-resolution mass spectrometer. Of these, four sites correspond to N-terminal acetylation identified in our study, with an additional site reported previously, whereas only five acetylation sites correspond to lysine acetylation (Table 1).

To assign novel modified residues, we compared the sites identified here against known PTMs in publicly available databases. We examined the UniProtKB (www.uniprot.org), PHOSIDA (www.phosida.com) and PTMfunc (www.ptmfunc.com) databases. While UniProtKB stores functional information on proteins such as biological ontologies, classifications and cross-references, PHOSIDA and PTMfunc are more specific and list PTMs of particular proteomes or proteins. Out of the 20 phosphosites determined in eIF2 and eIF2B, 6 sites have not been reported previously. The number of newly identified acetylation sites is more incisive: eight out of nine acetylation sites identified in this study represent novel modified sites.

### Location of PTMs

**Figure 4:**
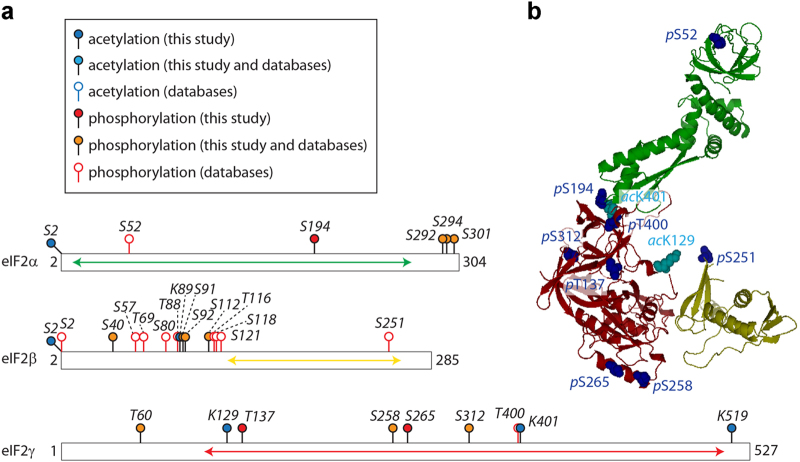
Location of identified post-translational modifications (PTMs) in eukaryotic initiation factor 2 (eIF2). (**a**) Phosphosites and acetylation sites identified in this or previous studies are shown on the protein subunits of eIF2. The proteins are represented as bars. The arrows (green, yellow and red) represent the parts of the sequence for which homology models could be obtained. (**b**) Homology models of eIF2-α, -β and -γ aligned with the archaeal homologue (PDB ID 3CW2). Phosphosites (dark blue) and acetylation sites (cyan) are shown as space-filled spheres. Please note that S52 is usually referred to as S51 in literature, which derives from corrected residue numbering after removal of N-terminal methionine. The numbering used here includes N-terminal methionine.

**Figure 5:**
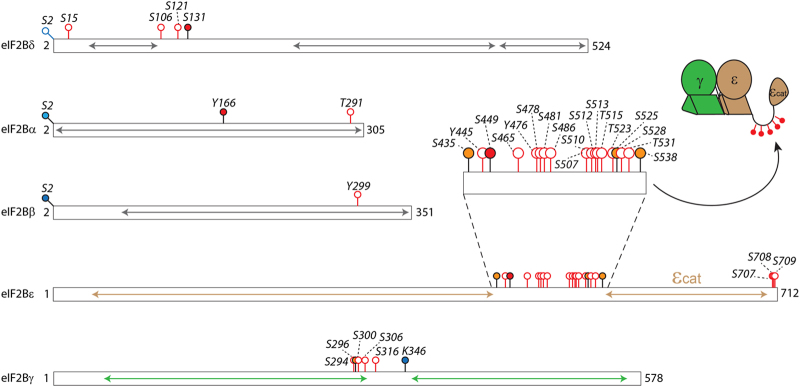
Location of post-translational modifications (PTMs) in eukaryotic initiation factor 2B (eIF2B). Phosphosites and acetylation sites identified in this or previous studies are shown on the protein subunits of eIF2. The proteins are represented as bars. The arrows represent the parts of the sequences for which homology models could be obtained. The magnification shows a phosphosite cluster of eIF2Bε. This cluster is located in a flexible loop connecting the ε/γ dimer (cartoon insert) and the catalytic domain of eIF2Bε.

To visualise these PTMs, we used a bar representation of the protein sequences and highlighted PTMs identified here and those present in databases. Our results show that phosphorylation is prevalent compared with acetylation, and all eIF2 and eIF2B subunits are phosphorylated at least once (Figures 4 and 5, and Table 1). Surprisingly, most of the phosphosites cluster in close proximity; for instance, we identified phosphorylation on residues S292, S294 and S301 of eIF2α and S91, S92 and S112 of eIF2β (Figure 4 and Table 1). Similarly, eIF2Bε appears to be only phosphorylated in the C-terminal half of the protein (all phosphosites being located on residues >435; Figure 5). This is in good agreement with available information from the databases, which provide additional phosphorylated residues in the clusters identified here (Supplementary Table S7).

Phosphorylation in eIF2 mainly occurs at the C-terminus of α-subunit and the N-terminal half of β-subunit. Phosphosites in eIF2γ are more evenly distributed throughout the protein (Figure 4a). Of the six acetylation sites identified in eIF2, two are located at the N-termini of the α- and β-subunits. The other sites are located in the β- and γ-subunits. The clustering as described for the phosphosites is not observed for acetylation. It is noteworthy, however, that the acetylation sites are often located in close proximity to phosphorylated residues (Figure 4a).

We next considered the location of phosphosites and acetylation sites on the homology model of eIF2. As the subunit sequences are not fully covered in the homology models of the protein subunits (see above), we could only locate 10 PTMs on eIF2. The majority of these PTMs are located in the Met-tRNA_i_^Met^ binding cleft suggesting their importance for Met-tRNA_i_^Met^ or nucleotide binding. A number of phosphosites are also located at eIF2 subunit interfaces implying their role in modulating the stability of the protein complex (Figure 4b). Interestingly, the unstructured N-terminal region of eIF2β (residues 40–121) is a region of high phosphorylation and likely regulates its proposed interactions with eIF5 [30].

Displaying the PTMs identified in eIF2B subunits shows that phosphorylation is mainly found in the catalytic γ- and ε-subunits. In both subunits, clusters of phosphosites are observed with PTMs in other subunits being less frequent (Figure 5). Most strikingly, a region that is highly phosphorylated was observed in a flexible linker of the ε-subunit. This linker connects the catalytic domain of eIF2Bε with the ε/γ dimer. No homology models could be obtained for this part of the ε-subunit [9], suggesting its extreme flexibility. The presence of PTMs in this flexible linker implies that this particular region of the ε-subunit is the key to control interactions between the catalytic domain of eIF2Bε and eIF2, thereby regulating the nucleotide exchange mechanism (Figure 5).

## Discussion

In this study, we characterised eIF2 and the ternary complex eIF2:GTP:Met-tRNA_i_^Met^, both representing important checkpoints for translation initiation in eukaryotes. We used complementary MS techniques to gain insights into conformational changes that occur in response to Met-tRNA_i_^Met^ binding. MS of the intact protein complex confirmed the 1:1:1 stoichiometry of all three protein subunits, and revealed that the β-subunit is only weakly associated. Stable α/γ dimers have been described in previous studies [5, 31]. Chemical cross-linking further showed that eIF2 is highly flexible in solution, allowing the α- and β-subunits to make contact across the binding site in the absence of Met-tRNA_i_^Met^.

Structural alignment of the homology models with the archaeal crystal structure of eIF2 reveals an elongated arrangement of the three protein subunits with the γ-subunit forming the centre of this initiation factor. In a previous study, we showed that interactions with the nucleotide exchange factor eIF2B are mediated via this elongated conformation bringing the nucleotide binding site of eIF2γ in close proximity to the catalytic domain of eIF2Bε [9]. After binding Met-tRNA_i_^Met^, we identified a conformational change of eIF2 that transforms the complex into a more compact arrangement. eIF2 flexibility is then impeded and the ternary complex adopts a more rigid structure with Met-tRNA_i_^Met^ bound. Comparative cross-linking allowed us to show that interactions between the α- and β-subunits are significantly reduced in the ternary complex owing to the occupancy of Met-tRNA_i_^Met^ in the cavity of eIF2.

**Figure 6:**
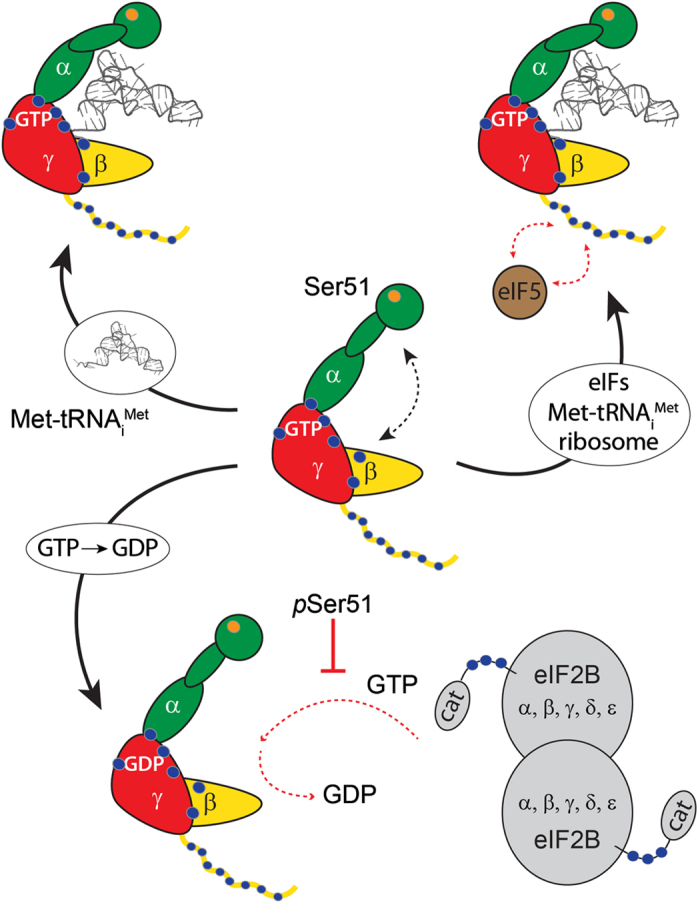
Eukaryotic initiation factor 2 (eIF2) interactions with initiator tRNA and eIF2B are regulated by post-translational modifications (PTMs) and conformational dynamics. Cartoon representation of eIF2 (α, green; β, yellow; and γ, red), Ser51 is highlighted (orange). Locations of PTMs (phosphorylation and acetylation) are indicated (blue; see also Figure 4b). Interactions stabilised by PTMs are highlighted (blue). GTP-bound eIF2 is highly flexible in solution. After binding of Met-tRNA_i_^Met^, the ternary complex adopts a more rigid conformation. The unstructured N-terminus of GTP-bound eIF2β interacts with eIF5. Met-tRNA_i_^Met^ release from eIF2 and transfer to the ribosome is triggered by GTP hydrolysis. GDP is exchanged by nucleotide exchange factor eIF2B preparing eIF2 for a new round of translation initiation. Nucleotide exchange is inhibited by phosphorylation of Ser51.

Our findings of the different conformations of eIF2 in the various stages of its cycle suggest that eIF2 adopts conformations according to its functional roles (Figure 6). The flexibility of apo eIF2 in solution keeps the complex in a form that can readily adopt different functional states. An important step for eIF2 is nucleotide exchange by eIF2B after release from the ribosome. This event has been shown previously to be affected by phosphorylation of eIF2α causing tight binding of the two eIFs [21]. The high levels of phosphorylation described for eIF2Bε, between the catalytic domain and the core of the complex, a region implicated in interactions with eIF2, strongly implies a functional relevance in which multiple modified residues in close proximity have a role in stabilising or destabilising protein interactions [29]. A further critical interaction of eIF2 is its contact with other initiation factors, specifically interactions of the N-terminal domain of eIF2β with eIF5 have been reported [30, 32]. Interestingly, the same N-terminal region of eIF2β has also been reported to interact with the catalytic domain of eIF2Bε during nucleotide exchange. Thus, this N-terminal region of eIF2β represents a competing target for protein interactions during translation initiation and its control [32]. In this regard, our observation of a highly phosphorylated N-terminus of eIF2β implies that regulation of these alternate interaction pathways may be mediated through different PTMs.

It is important to point out, however, that proteomic studies usually present an average of modified residues, their degree of modification being rarely 100%. The different positions and abundances of these modifications in eIF2/eIF2B could therefore represent alternative modification sites with the same or similar functional roles. Moreover, it is established that phosphorylation in eukaryotic cells is highly dynamic and dependent on the cell cycle phase. A number of regulated phosphosites was identified from large-scale studies, and kinase-motif analysis revealed that most of these sites are substrates for cell-cycle-dependent kinases [33, 34]. Our findings that both eIF2 and eIF2B are highly phosphorylated with several modified residues per protein subunit are in accord with the major role of phosphorylation being the regulation of cellular events. Translation initiation is a highly regulated process, and the phosphosites located here in all likelihood represent regulatory sites. Interestingly, the proportion of phosphorylated serine (85%), threonine (10%) and tyrosine (5%) residues agrees well with the results of other protein systems [35], wherein 87% of all phosphosites that are regulated during the cell cycle are on serine residues, whereas only 12% and 1% of the regulated sites are on threonine and tyrosine residues, respectively [35].

Questioning the relevance of the phosphorylated residues identified here, we investigated their conservation in different species. We examined the PTMfunc database and compared our phosphosites against different species. We found that 10 of our 20 phosphosites are conserved in *Homo sapiens, Mus musculus, Caenorhabditis elegans, Drosophila melanogaster, Candida albicans* or *Schizosaccharomyces pombe* with six sites being conserved in yeast *Schizosaccharomyces pombe*, two in *Candida albicans* and two in *Homo sapiens*. This high level of conservation further indicates their functional or structural importance in coordinating interactions. In some cases, a functional role has already been assigned; for instance, phosphorylation of Ser525 in eIF2Bε has been found to be crucial for eIF2B regulation [36].

In contrast to phosphorylation, the role of acetylation in the cell is not yet fully understood. Nonetheless, the acetylome of eukaryotes and bacteria is gaining attention with the discovery of acetylation sites in metabolic enzymes, ribosomes and chaperones from compete cell lysates [37–39] (for detailed review, see Choudhary *et al*. [40]). A regulatory role of acetylation in yeast *Saccharomyces cerevisiae* and conservation of a large fraction of acetylated lysine residues have also been described [41]. In the eight eIF2/eIF2B subunits investigated here, we identified several acetylation sites; five lysines and three N-termini were found to be acetylated and have not been reported before (Supplementary Table S7). Rather than having a regulatory effect, N-terminal acetylation of proteins has recently been described as a signal for degradation or prevention of degradation [42]. We speculate that the acetylation of the N-termini uncovered here could have a role in stabilising eIFs for complex formation during translation initiation.

The overall picture that emerges from this study is one of a coordinated series of events in which the highly flexible eIF2 fine-tunes interactions with Met-tRNA_i_^Met^, with other initiation factors and with the nucleotide exchange factor eIF2B adapting its structure and dynamics, as well as its phosphorylation status accordingly for each task. The regions of phosphorylation identified here and their location implies that they likely mediate interactions between subunits within eIF2, with eIF2B and other eIFs or with Met-tRNA_i_^Met^. Two long unstructured regions of eIF2Bε and eIF2β were found to be highly phosphorylated. By contrast, large regions of the eIF2B γ/ε subunits have no modifications, thereby suggesting limited access to kinases and acetylases.

Bringing together these results in a structural and dynamical context suggests that protein interactions within eIF2 and its conformational changes are mediated and controlled by many PTMs. The complexity that arises from the multitude of possible combinations of PTMs makes understanding the effects of individual modifications particularly challenging. It is clear, however, that key interaction interfaces have high levels of modification, and that eIF2 undergoes significant conformational change upon Met-tRNA_i_^Met^ binding. As such, our findings prompt new avenues of research to identify the kinases and acetylases responsible for key modifications and allow new insight into possible mechanisms of regulation and control of eIF2 interactions.

## Materials and Methods

### Purification of eIF2

His-tagged eIF2 was purified from *Saccharomyces cerevisiae* strain GP3511 (MATα *leu2-3, -112 ura-52 inol gcn2Δ pep4::LEU2 sui2Δ HIS4-lacZ* pAV1089 [*SUI2 SUI3 6xHisGCD22 URA3])* using Ni- and heparin-affinity chromatography as described [11].

Briefly, cells were grown in YPD media and lysed with two passes through a microfluidizer at 23 000 psi (M-110PS, microfluidics). The supernatant was mixed with nickel resin (GE Healthcare, Cleveland, OH, USA) and agitated on ice for 1 h. Bound protein was eluted stepwise with 15  and 100 mM EDTA. Eluted fractions were subjected to gel electrophoresis and eIF2 containing fractions were pooled for further purification.

eIF2 containing fractions were loaded on a heparin column. Bound eIF2 was eluted using a gradient ramp up to 1 M KCl over 20 column volumes. Eluted fractions were subjected to gel electrophoresis and eIF2 containing fractions were dialysed overnight against 38 mM HEPES (pH 7.4), 3.25 mM MgCl_2_, 5 mM β-Mercaptoethanol (β-ME) and 135 mM KCl.

### Purification of eIF2B

FLAG-tagged eIF2B was purified from yeast *Saccharomyces cerevisiae* strain GP4109 (*MAT*α *leu2-3 leu2-112 ura3-52 ino1 gcd6*Δ *gcn2*Δ::hisG *ura3-52::HIS4-lacZ* pAV1428[*GCD6 GCD1*-FLAG2-His6 *URA3* 2 μm] pAV1494[*GCN3 GCD2 GCD7 LEU2* 2 μm]) [43] as described [44].

Briefly, FLAG-tagged eIF2B complex was purified in a single affinity step in a high-salt buffer (500 mM KCl). After immobilizing the protein on anti-FLAG resin, the protein was washed with 500 mM ammonium acetate and eluted with FLAG peptide. The eluted fraction (~400 μl) was concentrated in an Amicon Ultra (100 K molecular weight cutoff) centrifugal filter (Millipore, Billerica, MA, USA).

### Purification of Met-tRNA_i_^Met^

Met-tRNA_i_^Met^ was obtained from yeast tRNA type X (Sigma-Aldrich, St Louis, MO, USA) by aminoacylation using aminoacyl-tRNA synthetase from *Escherichia coli* (Sigma-Aldrich) as described previously [21]. Fifty units per ml yeast tRNA type X were incubated for 60 min at 37 °C with 100 μM methionine and 100 μg ml^−1^ aminoacyl-tRNA synthetase in 300 μl 150 mM HEPES (pH 7.0), 15 mM MgCl_2_, 1 mg ml^−1^ bovine serum albumin and 100 μM ATP. The mixture of tRNAs was further purified by phenol/chloroform extraction and ethanol precipitation following standard protocols.

### Formation of the ternary complex

The ternary complex was assembled as described [45] by incubating an excess of tRNAs with eIF2 in 38 mM HEPES (pH 7.4), 3.25 mM MgCl_2_, 5 mM β-mercaptoethanol, 100 mM KCl, 0.4 mM GDPNP (Sigma-Aldrich) and 1 mM ATP for 30 min at room temperature. Only Met-tRNA_i_^Met^ specifically binds eIF2. The purified ternary complex was separated from nucleotide, methionine and tRNAs by filtration using an Amicon filtration device (Millipore, Billerica, MA, USA) with molecular weight cutoff of 30 kDa.

### MS of intact protein complexes

Before MS analysis, the purification buffer was exchanged against 500 mM ammonium acetate using an Amicon filtration device with molecular weight cutoff of 30 kDa. Mass spectra were acquired on a QSTAR XL mass spectrometer, Framingham, MA, USA modified for transmission of high-mass complexes [22, 46] using gold-coated glass capillaries prepared in-house [47]. Instrument parameters were varied as follows: ion spray voltage 1.3 kV, declustering potential 50–150 V, focusing potential 100–200 V and collision energy up to 200 V, MCP 2 350 V. For MS/MS, the relevant *m/z* range was selected in the second quadrupole and subjected to high collision cell energy.

### Homology modelling

Homology models for yeast eIF2 α-, β- and γ-subunits were generated using MODELLER [48] (https://modbase.compbio.ucsf.edu/scgi/modweb.cgi) and SWISS-MODEL [49] (http://swissmodel.expasy.org/) web servers.

### Protein–protein cross-linking

Protein complexes were incubated with a 1:1 mixture of 2.5 mM deuterated and non-deuterated bis(sulfosuccinimidyl)suberate (BS3-d0 and BS3-d4; Thermo Scientific) for 1 h at room temperature and 350 r.p.m. in a thermomixer. The cross-linked protein samples were subsequently loaded onto a NuPAGE gel (Invitrogen, Carlsbad, CA, USA), and protein bands were cut for digestion with Trypsin.

### Comparative cross-linking

Comparative cross-linking was performed as described [26]. Two aliquots of the protein complex were treated either as the control or subjected to Met-tRNA_i_^Met^ binding (see above) and cross-linked separately with BS3-d0 and BS3-d4. Subsequently, equal volumes or concentrations were pooled in a 1:1 ratio. The pooled sample was then loaded onto a NuPAGE gel and protein bands were excised for tryptic digestion.

### Protein digestion

Gel-separated proteins were digested in-gel using Trypsin (Roche, Indianapolis, IN, USA) as described previously [50]. For PTM analysis, proteins were also digested in solution using RapiGest SF Surfactant (Waters, Milford, MA, USA) according to manufacturer’s protocols.

### Phosphopeptide enrichment

Phosphopeptides were enriched using TiO_2_. Columns were packed into pipette tips using TiO_2_ material (GL Sciences, Tokyo, Japan). The TiO_2_ material was washed with 5% Trifluoroacetic acid (TFA)/80% acetonitrile (ACN) and reconstituted in 20% 2,5-dihydroxybenzoic acid/5% TFA/80% ACN. Peptides were dissolved in 20% dihydroxybenzoic acid/5% TFA/80% ACN and loaded onto the material. After washing with 20% dihydroxybenzoic acid/5% TFA/80% ACN and 5% TFA/80% ACN, phosphopeptides were eluted with 0.3 N ammonia solution (pH >10.5). Eluted peptides were dried in a vacuum centrifuge and stored for LC-MS/MS analysis.

### LC-MS/MS analysis for protein identification, phosphosite analysis after enrichment of phosphopeptides and cross-linking

Tryptic peptides were separated by nano-flow reversed-phase liquid chromatography using a DionexUltiMate 3000 RSLC nano system (Thermo Scientific, Waltham, MA, USA). The peptides were loaded onto a trap column (HPLC column Acclaim PepMap 100, C18, 100 μm inner diameter particle size 5 μm; Thermo Scientific) and separated with a flow rate of 300 nl min^−1^ on an analytical C18 capillary column (50 cm, HPLC column Acclaim PepMap 100, C18, 75 μm inner diameter particle size 3 μm; Thermo Scientific), with a gradient of 5–80% (v/v) mobile phase B over 74 min (mobile phase A:0.1% (v/v) formic acid; mobile phase B: 80% (v/v) ACN/0.1% (v/v) formic acid). Peptides were eluted directly into an LTQ-Orbitrap XL hybrid mass spectrometer (Thermo Scientific).

MS conditions were as follows: spray voltage of 1.8 kV; capillary temperature of 180 °C; normalised collision energy of 35% at an activation of *q*=0.25; and an activation time of 30 ms. The LTQ-Orbitrap XL was operated in data-dependent mode. Survey full-scan spectra were acquired in the orbitrap (*m/z* 300–2 000) with a resolution of 30 000 at *m/z* 400 and an automatic gain control target at 10^6^. The five most intense ions were selected for CID MS/MS fragmentation in the linear ion trap at an automatic gain control target of 30 000. Detection in the linear ion trap of previously selected ions was excluded dynamically for 30 s. Singly charged ions as well as ions with unrecognised charge state were also excluded. The orbitrap mass analyser was internally calibrated using the lock mass option (lock mass: *m/z* 445.120025 [51]).

### LC-MS/MS analysis for identification of PTMs using a Q Exactive Orbitrap mass spectrometer

Proteins were digested as described in Shevchenko *et al.* [50]. Peptides were separated by nano-flow reversed-phase liquid chromatography (EASY nLC 1 000, Thermo Scientific; mobile phase A, 0.1% (v/v) formic acid/5% (v/v) dimethyl sulphoxide; mobile phase B, 100% (v/v) ACN/0.1% (v/v) formic acid/5% (v/v) dimethyl sulphoxide) coupled to a Q Exactive Orbitrap mass spectrometer (Thermo Scientific). Peptides were loaded onto a trap column (5 mm, PepMap RSLC, C18, 300 μm inner diameter particle size 3 μm; Thermo Scientific) and separated with a flow rate of 200 nl min^−1^ on an analytical C18 capillary column (50 cm, PepMap RSLC, EASY-spray column, C18, 75 μm inner diameter particle size 3 μm; Thermo Scientific), with a gradient of 7–30% (v/v) mobile phase B over 30 min. Column temperature was 40 °C. Peptides were directly eluted into the mass spectrometer.

MS conditions were as follows: spray voltage of 2.1 kV and capillary temperature of 320 °C. The Q Exactive Orbitrap was operated in data-dependent mode. Survey full-scan MS spectra were acquired in the orbitrap (*m*/*z* 350−1 500) with a resolution of 70 000 an automatic gain control target at 3×10^6^. The 10 most intense ions were selected for Higher energy collisional dissociation (HCD) at an automatic gain control target of 50 000.

### Database search for protein identification and analysis of PTMs

The raw data was converted to mgf file format using MassMatrix file conversion tool (www.massmatrix.net). Mgfs were searched against Swiss-Prot (August 2014; 546 238 sequences) or NCBInr (December 2013; 34 397 589 sequences) databases using Mascot search engine v2.4.1 (MatrixScience). The taxonomy filter *Saccharomyces cerevisiae* filter was enabled and the mass accuracy filter was 10 p.p.m. for precursor ions and 0.6 Da for fragment ions for data acquired on an Orbitrap-XL mass spectrometer, and 7 p.p.m. and 0.01 Da, respectively, for data acquired on a Q Exactive Orbitrap mass spectrometer. Peptides were defined to be tryptic with a maximum of two missed cleavage sites. Carbamidomethylation of cysteine and oxidation of methionine residues was allowed as variable modification. For identification of PTMs, phosphorylation on serine, threonine and tyrosine, as well as acetylation on lysine and the protein N-terminus were also allowed.

### Database search of cross-linked peptides and generation of extracted ion chromatograms

Potential cross-links were identified by using the MassMatrix Database Search Engine [52] using the following search parameters: maximum of two missed tryptic cleavage sites, carbamidomethylation of cysteine and oxidation of methionine residues as variable modifications, mass accuracy filter of 10 p.p.m. for precursor ions and 0.8 Da for fragment ions, minimum pp and pp_2_ values of 5.0, minimum pp_tag_ of 1.3 and maximum number of cross-links per peptide=1.

Two database searches were performed for (i) deuterated and (ii) non-deuterated cross-linked peptides, respectively. Potential cross-links were validated by (i) the presence of the d4/d0-BS3 generated peak pair in the MS spectra and (ii) by the quality of the MSMS spectrum assessed manually.

Extracted ion chromatograms were generated separately for light (d0) and heavy (d4) peaks of the peak pair using XCalibur software v2.1 (Thermo Scientific). Cross-linking ratios were calculated from the peak area of the extracted ion chromatograms.

## Figures and Tables

**Table 1:** Summary of PTMs identified in this study

*Protein subunit*	*Modified residue*	*Highest Mascot score*	*TiO*_*2*_ *enrichment*	*Direct analysis*	*Database*	*Conservation*
eIF2α	*N-term-ac-*S2	46		×	+	
	*p*S194	16	×		+	
	*p*S292	24		×	A, B, C	*S. pombe*
	*p*S294	17		×	A, B, C	*S. pombe, C. elegans*
	*p*S301	42		×	B, C	*C. elegans*
eIF2β	*N-term-ac-*S2	63		×	+	
	*p*S40	76	×	×	A, B, C	*S. pombe, C. albicans*
	*ac*K89	49		×	+	
	*p*S91	39	×		C	*S. pombe*
	*p*S92	43	×	×	A, C	*S. pombe*
	*p*S112	64	×	×	A, B, C	*D. melanogaster*
eIF2γ	*p*T60	88	×		A, C	
	*ac*K129	37		×	+	
	*p*T137	16	×		+	
	*p*S258	41	×		A, C	
	*p*S265	27	×		+	
	*p*S312	32	×		C	*C. albicans*
	*ac*K401	17		×	+	
	*ac*K519	47		×	+	
eIF2Bα	*N-term-ac-*S2	52		×	A	
	*p*Y166	19	×		+	
eIF2Bβ	*N-term-ac-*S2	79		×	+	
eIF2Bγ	*p*S296	39	×		A, C	
	*ac*K346	58		×	+	
eIF2Bδ	*p*S131	49	×		+	
eIF2Bε	*p*S435	66	×		C	
	*p*S449	72	×		+	
	*p*S525	67	×	×	A, C	*H. sapiens*
	*p*S538	57	×	×	A, B, C	*H. sapiens, M. musculus, S. pombe*

Abbreviations: *ac*, acetylation; eIF2, eukaryotic initiation factor 2; *p*, phosphorylation; PTMs, post-translational modifications; TiO_2_, titanium dioxide.

Protein subunits of eIF2 and eIF2B and their modified residues (*p* and *ac*) with the highest observed Mascot score for each site are listed. It is indicated whether the sites were determined after TiO_2_ enrichment or by direct analysis. For PTMs that have been reported before the respective databases are given (A, UniProtKB; B, PHOSIDA; and C, PTMfunc).

+ Indicates novel sites. Note that acetylation sites have only been analysed without enrichment.
