# Supplementary material for: eIF2 interactions with initiator tRNA and eIF2B are regulated by post-translational modifications and conformational dynamics
Source: Cell Discov. 2015 Aug 11;1:15020–. doi: 10.1038/celldisc.2015.20 (PMC4860841; doi:10.1038/celldisc.2015.20)
Supplement: Supplementary Information [file celldisc201520-s1.doc]

**Supplementary Information**

**eIF2 interactions with initiator tRNA and eIF2B are regulated by**

**post-translational modifications and conformational dynamics**

Victoria Beilsten-Edmands^1^, Yuliya Gordiyenko^1,3^, Jocky C. K. Kung^1,4^, Shabaz Mohammed^1,2^, Carla Schmidt^1*^, and Carol V. Robinson^1*^

*joint corresponding authors

^1^Department of Chemistry, University of Oxford, South Parks Road, Oxford, United Kingdom.

^2^Department of Biochemistry, University of Oxford, South Parks Road, Oxford, United Kingdom.

^3^present address: MRC Laboratory of Molecular Biology, Francis Crick Avenue, Cambridge, United Kingdom

^4^present address: Department of Chemistry, University of Toronto, Saint-George Street, Toronto, Ontario, Canada

**Figures**

**
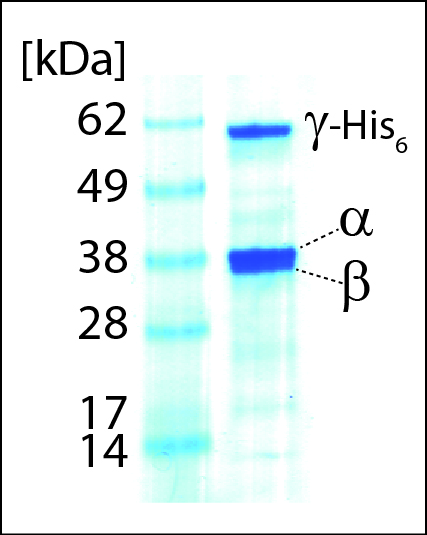
**

**Figure S1: SDS-PAGE of eIF2.** His-tagged eIF2 was purified from yeast. SDS-PAGE and LC-MS/MS confirmed the presence of three protein subunits (see also Table S1).


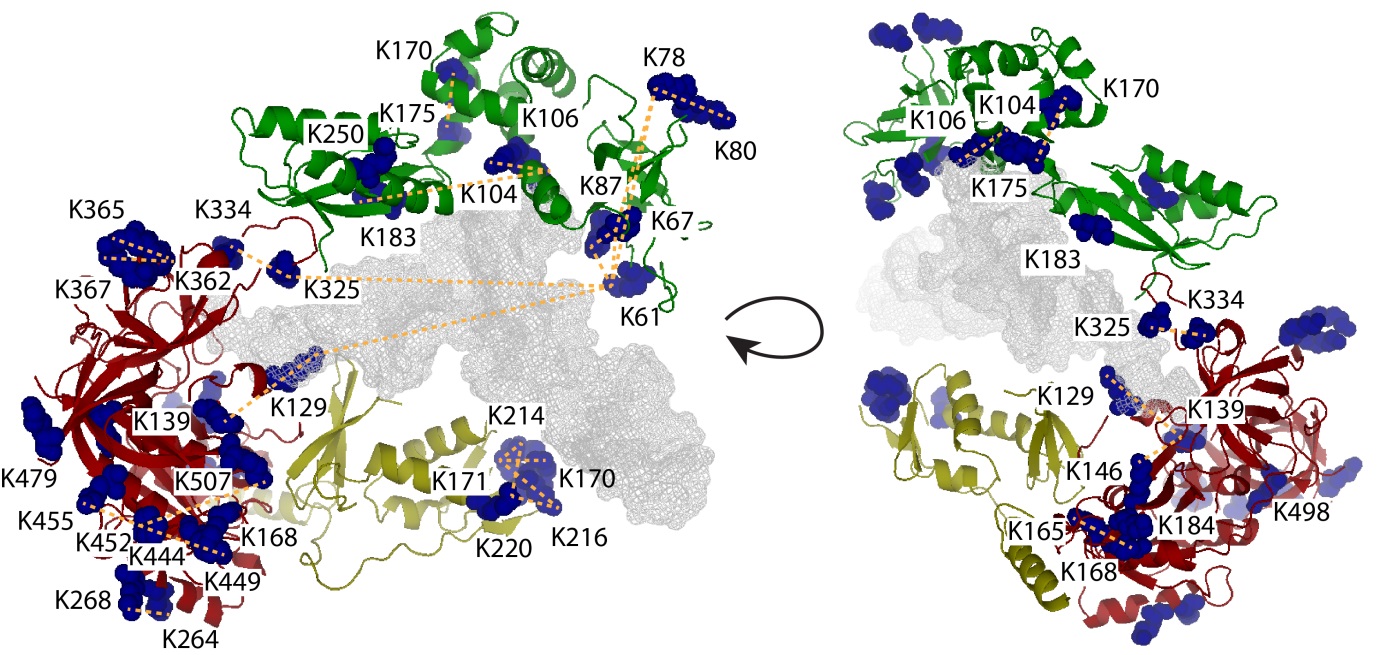


Figure S2: Cross-linking of the ternary complex. Homology models were aligned with the archaeal ternary complex homologue (PDB ID 3V11). Cross-linked residues are shown as blue space fillings and residue numbers are given. Identified cross-links are indicated as dotted lines (orange).


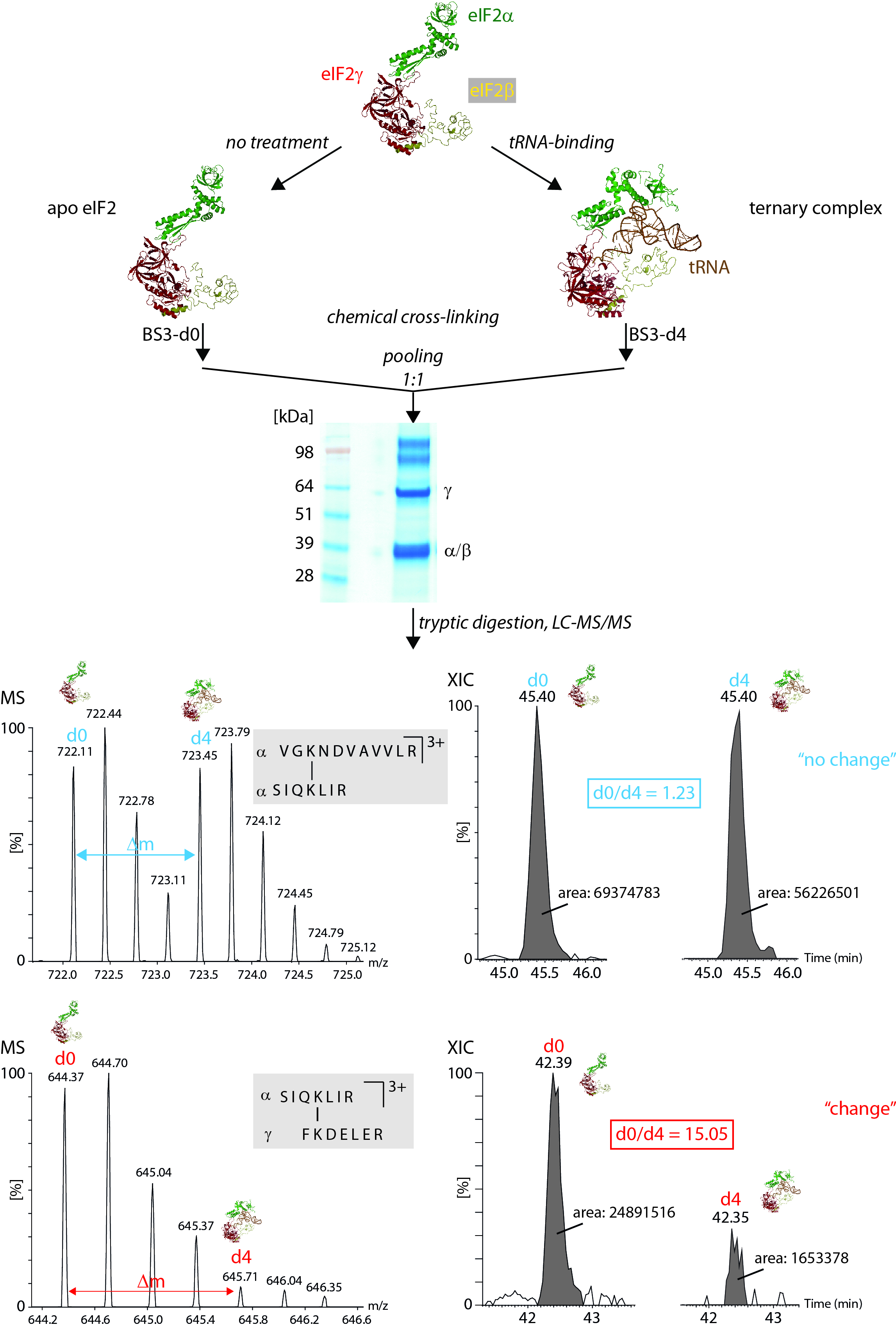


Figure S3: Comparative cross-linking workflow. eIF2 was incubated with tRNA to form the ternary complex, while a control sample was incubated with buffer (apo eIF2). Apo eIF2 and the ternary complex were cross-linked with BS3-d0 and BS3-d4, respectively. The two cross-linked protein complexes were pooled in the same amounts, separated by gel electrophoresis and analysed by LC-MS/MS. Protein interactions that did not change upon Met-tRNA_i_^Met^-binding showed equal intensities in MS spectra, while reduced interactions showed decreased intensities. Extracted ion chromatograms (XICs) were generated for the deuterated and non-deuterated cross-linked peptides and quantitative ratios were obtained from the peak areas of the XICs.


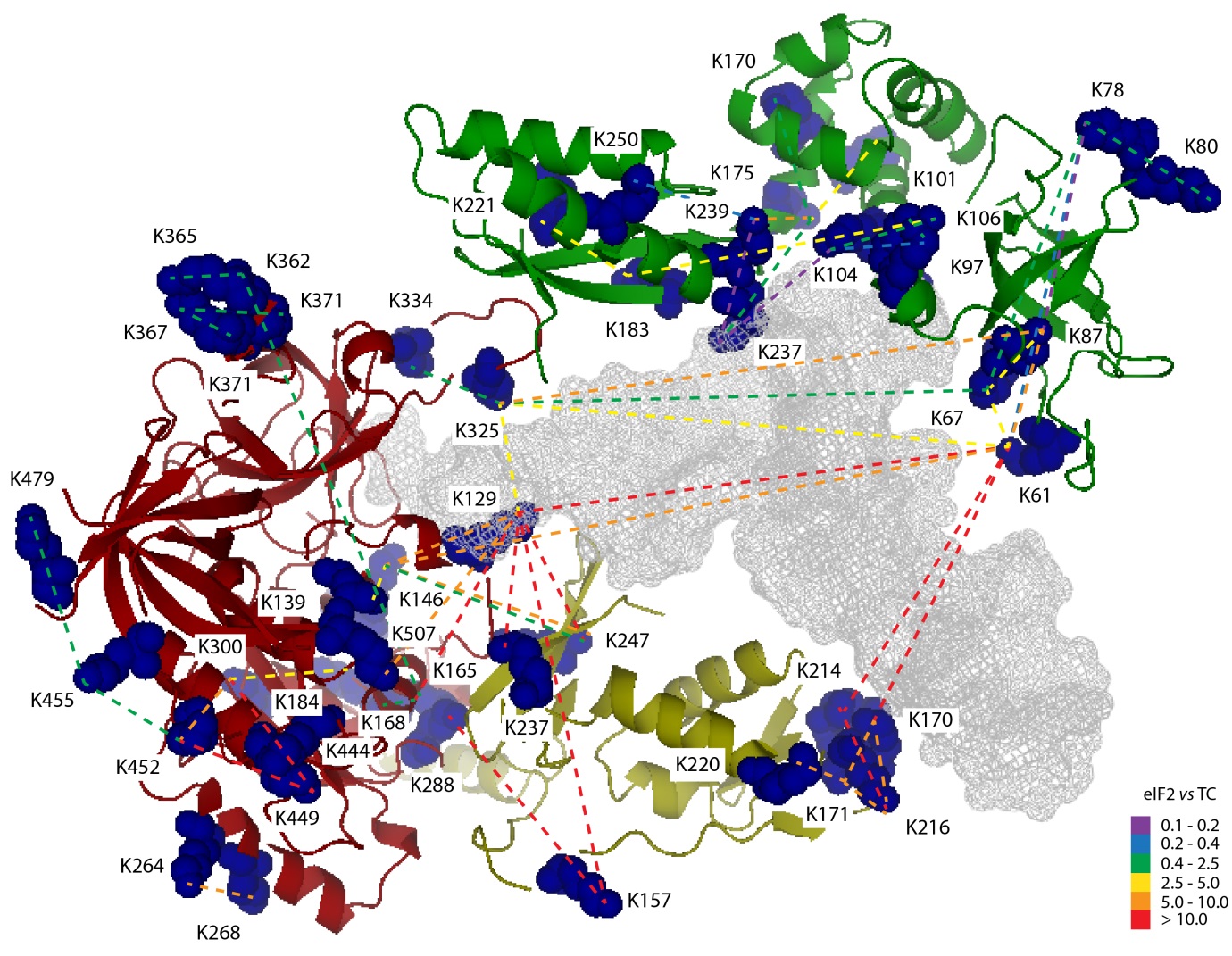


Figure S4: Comparative cross-linking of apo eIF2 vs the ternary complex. Homology models were aligned with the archaeal ternary complex homologue (PDB ID 3v11). Cross-linked residues are shown as blue space fillings and residue numbers are given. Identified cross-links are indicated with dotted lines. Changes in cross-linking intensities (eIF2/TC) are colour coded (see legend). Ratio of 1 means equal intensities in both eIF2 and TC, ratio > 1 means higher intensities in eIF2 and ratio < 1 means higher intensities in TC.

**Tables**

Table S1: Protein identification. Proteins were identified after in-gel digestion, LC-MS/MS and database searching. For each protein subunit the SWISS-Prot ID, the number of obtained spectra and sequences as well as the sequence coverage are given.

| Subunit | SWISS-Prot ID | # Spectra | # Peptide sequences | Sequence Coverage [%] |
| --- | --- | --- | --- | --- |
| γ | IF2G_YEAST | 384 | 74 | 75 |
| α | IF2A_YEAST | 283 | 63 | 93 |
| β | IF2B_YEAST | 286 | 58 | 94 |

Table S2: Masses of eIF2 subunits and (sub-) complexes. The theoretical and observed masses are given in kDa. For some sub-complexes two masses corresponding to bound/unbound GDP are given (molecular weight GDP: 443 Da).

| **Subunit / complex** | **Theoretical mass [kDa]** | **Observed mass [kDa]** |
| --- | --- | --- |
| eIF2α | 34.718 | 34.871 |
| eIF2β | 31.574 | 31.765 |
| eIF2β (stripped MS/MS) | - | 31.575 |
| eIF2γ-His_6_ | 59.150 / 58.707 | 59.369 / 58.950 |
| eIF2α/γ-His_6_ | 93.868 / 93.425 | 94.288 / 93.843 |
| eIF2α/γ-His_6_ (stripped MS) | - | 94.378 / 93.926 |
| eIF2α/γ-His_6_ (stripped MS/MS) | - | 93.575 |
| eIF2β/γ-His_6_ | 90.724 / 90.281 | 91.758 / 91.231 |
| eIF2β/γ-His_6_ (stripped MS/MS) | - | 91.025 |
| eIF2 (GDP-bound) | 125.442 | 126.135 |

Table S3: Cross-linking of eIF2. The cross-linked protein subunits, the sequences of the cross-linked peptides, the cross-linked amino acid residues, and the number of identified MSMS spectra are given for every cross-link. Cross-linked amino acid residues are highlighted in red, modified amino acids residues are labelled with * (C → carbamidomethylation, M → oxidation). In total 73 different cross-links have been identified; these include 26 inter-protein cross-links, 32 intra-protein cross-links and 15 inter-peptide cross-links. Cross-links highlighted in grey could not be assigned unambiguously.

| **Protein 1** | **Protein 2** | **Sequence 1** | **Sequence 2** | **Residue 1** | **Residue 2** | **# Spectra** |
| --- | --- | --- | --- | --- | --- | --- |
| alpha | alpha | VG**K**NDVAVVLR | SIQ**K**LIR | 67 | 61 | 18 |
|  |  | VG**K**NDVAVVLR | GYIDLS**K**R | 67 | 87 | 2 |
|  |  | VG**K**NDVAVVLR | S**K**TVHSILR | 67 | 106 | 2 |
|  |  | GYIDLS**K**R | SIQ**K**LIR | 87 | 61 | 5 |
|  |  | S**K**TVHSILR | LTPQAV**K**IR | 106 | 183 | 1 |
|  |  | YC*AE**K**FQIPLEELYK | NYIS**K**R | 118 | 175 | 7 |
|  |  | V**K**LVAAPLYVLTTQALDK | LTPQAV**K**IR | 221 | 183 | 9 |
|  |  | LVAAPLYVLTTQALD**K**QK | NYIS**K**R | 237 | 175 | 1 |
|  |  | Q**K**GIEQLESAIEK | NYIS**K**R | 239 | 175 | 1 |
|  |  | VD**K**E**K**GYIDLSK |  | 78/80 |  | 3 |
| alpha | beta | SIQ**K**LIR | DG**K**K | 61 | 170 | 1 |
|  |  | SIQ**K**LIR | SVSSI**K**TGFQATVGK | 61 | 273 | 1 |
|  |  | SIQ**K**LIR | TGFQATVG**K**R | 61 | 281 | 2 |
|  |  | VD**K**EK | DG**K**K | 78 | 170 | 1 |
|  |  | VDKE**K**GYIDLSK | TGFQATVG**K**R | 78 | 281 | 1 |
|  |  | NYIS**K**R | TGFQATVG**K**R | 175 | 281 | 1 |
| alpha | gamma | SIQ**K**LIR | **K**R | 61 | 66 | 1 |
|  |  | SIQ**K**LIR | F**K**DELER | 61 | 129 | 8 |
|  |  | SIQ**K**LIR | NITI**K**LGYANAK | 61 | 139 | 1 |
|  |  | SIQ**K**LIR | SF**K**SDK | 61 | 165 | 1 |
|  |  | SIQ**K**LIR | IE**K**HWR | 61 | 507 | 1 |
|  |  | SIQ**K**LIR | RIE**K**HWR | 61 | 507 | 2 |
|  |  | VG**K**NDVAVVLR | F**K**DELER | 67 | 129 | 1 |
| alpha | gamma | VG**K**NDVAVVLR | SFDVN**K**PGAEIEDLK | 67 | 325 | 1 |
|  |  | V**K**LVAAPLYVLTTQALDK | SFDVN**K**PGAEIEDLK | 221 | 325 | 1 |
|  |  | SIQ**K**LIR | SFDVN**K**PGAEIEDLK | 323 | 325 | 2 |
| beta | beta | SGP**K**FR | **K**K | 157 | 18/19/50/51/52/53/83/84/85/86 | 1 |
|  |  | TIFSNIQDIAE**K**LHR | SGP**K**FR | 184 | 157 | 1 |
|  |  | TGFQATVG**K**R | DG**K**K | 281 | 170 | 1 |
|  |  | TC***K**SINTELK | SGP**K**FR | 329 | 157 | 1 |
|  |  | TCKSINTEL**K**R | SGP**K**FR | 336 | 157 | 1 |
|  |  | DG**KK**TIFSNIQDIAEK |  | 170/171 |  | 5 |
|  |  | LVI**K**G**K**FQSK |  | 214/216 |  | 8 |
|  |  | **K**S**K**SVSADAEAEK |  | 54/56 |  | 1 |
|  |  | **K**T**K**DSSVDAFEK |  | 87/89 |  | 4 |
|  |  | T**K**DSSVDAFE**K**ELAK |  | 89/98 |  | 1 |
|  |  | DSSVDAFE**K**ELA**K**AGLDNVDAESK |  | 98/102 |  | 1 |
| beta | gamma | SGP**K**FR | **K**VAFTGLEEDGETEEEKR | 157 | 48 | 1 |
|  |  | SGP**K**FR | VAFTGLEEDGETEEE**K**RK | 157 | 64 | 1 |
|  |  | SGP**K**FR | F**K**DELER | 157 | 129 | 3 |
|  |  | DG**K**K | F**K**DELER | 170 | 129 | 1 |
|  |  | SGP**K**FR | **K**K | 157 | 47 | 1 |
|  |  | SGP**K**FR | SF**K**SDKEISPK | 157 | 165 | 2 |
|  |  | SINTEL**K**R | F**K**DELER | 247 | 129 | 1 |
|  |  | TGFQATVG**K**R | **K**R | 281 | 66 | 1 |
|  |  | TGFQATVG**K**R | F**K**DELER | 281 | 129 | 2 |
|  |  | **K**K | Y**K**LVR | 18/19/50/51/52/53/83/84/85/86 | 184 | 1 |
|  |  |  |  |  |  |  |
| gamma | gamma | **K**K | Y**K**LVR | 47 | 184 | 1 |
|  |  | VAFTGLEEDGETEEE**K**R | SD**K**EISPK | 64 | 168 | 1 |
|  |  | QATINIGTIGHVAHG**K**STVVR | F**K**DELER | 113 | 129 | 1 |
|  |  | QATINIGTIGHVAHG**K**STVVR | SF**K**SDK | 113 | 165 | 1 |
|  |  | F**K**DELER | SF**K**SDK | 129 | 165 | 4 |
|  |  | F**K**DELER | IE**K**HWR | 129 | 507 | 2 |
|  |  | NITI**K**LGYANAK | F**K**DELER | 139 | 129 | 9 |
|  |  | SF**K**SDK | **K**R | 165 | 66 | 5 |
|  |  | SF**K**SDKEISPK | **K**R | 165 | 66 | 7 |
|  |  | SF**K**SDKEISPK | F**K**DELER | 165 | 129 | 2 |
|  |  | SD**K**EISPK | **K**R | 168 | 66 | 4 |
|  |  | SFKSD**K**EISPK | F**K**DELER | 168 | 129 | 2 |
|  |  | SFDVN**K**PGAEIEDLK | F**K**DELER | 325 | 129 | 4 |
|  |  | LGDEIEIRPGIVT**K**DDK | F**K**DELER | 362 | 129 | 2 |
|  |  | LLGV**K**TDGQK | QA**K**VR | 444 | 452 | 1 |
|  |  | **K**LEPNEVLMVNIGSTATGAR | VVAV**K**ADM*AR | 455 | 479 | 7 |
|  |  | VVAV**K**ADM*AR | LIGWATI**K**K | 479 | 518 | 10 |
|  |  | VVAV**K**ADMAR | **K**GTTLEPIA | 479 | 519 | 8 |
|  |  | LGDEIEIRPGIVT**K**DD**K**GK |  | 362/365 |  | 2 |
|  |  | SF**K**SD**K**EISPK |  | 165/168 |  | 11 |
|  |  | EESALEHQ**K**SIL**K**FIR |  | 263/267 |  | 26 |
|  |  | SFDVN**K**PGAEIEDL**K**GGVAGGSILNGVFK |  | 325/334 |  | 2 |
|  |  | DD**K**G**K**IQC*KPIFSNIVSLFAEQNDLK |  | 365/367 |  | 4 |
|  |  | LLGV**K**TDGQ**K**QAK |  | 444/449 |  | 2 |
|  |  | TDGQ**K**QA**K**VR |  | 449/452 |  | 16 |
|  |  | **KK**VAFTGLEEDGETEEEK |  | 47/48 |  | 2 |

Table S4: Homology modelling of eIF2 subunits. These homology models were reported before. The employed server, the modelled segment, the PDB template, the sequence identity between the subunit and the template and the modelling score are given for eIF2 α, β and γ homology models.

| **Subunit** | **Server** | **Segment** | **PDB template** | **Sequence identity [%]** | **Score** |
| --- | --- | --- | --- | --- | --- |
| eIF2α | SWISS-MODEL | 7-267 | 3CW2 | 19.6 | 0.55 |
| eIF2β | SWISS-MODEL | 127-271 | 2QMU | 32.9 | 0.35 |
| eIF2γ | Modeller | 96-519 | 1KK1 | 48.0 | 1.00 |

Table S5: Cross-linking of the ternary complex. The cross-linked protein subunits, the sequences of the cross-linked peptides, the cross-linked amino acid residues, and the number of identified MSMS spectra are given for every cross-link. Cross-linked amino acid residues are highlighted in red, modified amino acids residues are labelled with * (C → carbamidomethylation, M → oxidation). In total 43 different cross-links have been identified; these include 2 inter-protein cross-links, 21 intra-protein cross-links and 20 inter-peptide cross-links. Cross-links highlighted in grey could not be assigned unambiguously.

| **Protein 1** | **Protein 2** | **Sequence 1** | **Sequence 2** | **Residue 1** | **Residue 2** | **# Spectra** |
| --- | --- | --- | --- | --- | --- | --- |
| alpha | alpha | VG**K**NDVAVVLR | GYIDLS**K**R | 67 | 87 | 1 |
|  |  | SIQ**K**LIR | VD**K**EK | 61 | 78 | 3 |
|  |  | VG**K**NDVAVVLR | SIQ**K**LIR | 67 | 61 | 8 |
|  |  | VG**K**NDVAVVLR | VD**K**EK | 67 | 78 | 4 |
|  |  | VD**K**EKGYIDLSK | SIQ**K**LIR | 78 | 61 | 1 |
|  |  | VDKE**K**GYIDLSK | VG**K**NDVAVVLR | 78 | 67 | 1 |
|  |  | GYIDLS**K**R | SIQ**K**LIR | 87 | 61 | 3 |
|  |  | S**K**TVHSILR | LTPQAV**K**IR | 106 | 183 | 1 |
|  |  | LVAAPLYVLTTQALD**K**QK | YQ**K**SK | 237 | 104 | 3 |
|  |  | LVAAPLYVLTTQALD**K**QK | NYIS**K**R | 237 | 175 | 1 |
|  |  | Q**K**GIEQLESAIEK | NYIS**K**R | 239 | 175 | 1 |
|  |  | YQ**K**S**K**TVHSILR |  | 104/106 |  | 1 |
|  |  | DVLDEL**K**NYIS**K**R |  | 170/175 |  | 2 |
|  |  | LVAAPLYVLTTQALD**K**Q**K**GIEQLESAIEK |  | 237/239 |  | 1 |
|  |  | Q**K**GIEQLESAIE**K**ITEVITK |  | 239/250 |  | 1 |
|  |  | VD**K**E**K**GYIDLSK |  | 78/80 |  | 5 |
| alpha | gamma | SIQ**K**LIR | SFDVN**K**PGAEIEDLK | 61 | 325 | 1 |
|  |  | SIQ**K**LIR | F**K**DELER | 61 | 129 | 3 |
| beta | beta | **K**TIFSNIQDIAEK | LVI**K**GK | 171 | 214 | 4 |
|  |  | FQS**K**QM*ENVLR | LVI**K**GK | 220 | 214 | 1 |
|  |  | DG**KK**TIFSNIQDIAEK |  | 170/171 |  | 9 |
|  |  | LVI**K**G**K**FQSK |  | 214/216 |  | 6 |
|  |  | G**K**FQS**K**QM*ENVLR |  | 216/220 |  | 4 |
| beta | beta | SVSSI**K**TGFQATVG**K**R |  | 272/281 |  | 1 |
|  |  | **K**T**K**DSSVDAFEK |  | 87/89 |  | 2 |
|  |  | T**K**DSSVDAFE**K**ELAK |  | 89/98 |  | 1 |
| gamma | gamma | VAFTGLEEDGETEEE**K**R | **K**R | 64 | 66 | 5 |
|  |  | NITI**K**LGYANAK | F**K**DELER | 139 | 129 | 1 |
|  |  | YNIDAVNEFIV**K**TIPVPPR | Y**K**LVR | 300 | 184 | 1 |
|  |  | LLGV**K**TDGQK | RIE**K**HWR | 444 | 507 | 1 |
|  |  | **K**LEPNEVLM*VNIGSTATGAR | QA**K**VR | 455 | 452 | 1 |
|  |  | VVAV**K**ADM*AR | LIGWATI**K**K | 479 | 518 | 7 |
|  |  | VVAV**K**ADMAR | **K**GTTLEPIA | 479 | 519 | 1 |
|  |  | LQLTSPAC*TEINE**K**IALSR | LIGWATI**K**K | 498 | 518 | 1 |
|  |  | NITI**K**LGYANA**K**IYK |  | 139/146 |  | 4 |
|  |  | SF**K**SD**K**EISPK |  | 165/168 |  | 9 |
|  |  | EESALEHQ**K**SIL**K**FIR |  | 264/268 |  | 11 |
|  |  | SFDVN**K**PGAEIEDL**K**GGVAGGSILNGVFK |  | 325/334 |  | 1 |
|  |  | LGDEIEIRPGIVT**K**DD**K**GK |  | 362/365 |  | 5 |
|  |  | DD**K**G**K**IQC*KPIFSNIVSLFAEQNDLK |  | 365/367 |  | 2 |
|  |  | LLGV**K**TDGQ**K**QAK |  | 444/449 |  | 4 |
|  |  | TDGQ**K**QA**K**VR |  | 449/552 |  | 15 |
|  |  | LIGWATI**KK**GTTLEPIA |  | 518/519 |  | 3 |

Table S6: Comparative cross-linking of the eIF2 *vs* the ternary complex. The cross-linked protein subunits, the sequences of the cross-linked peptides, the cross-linked amino acid residues, the number of identified MSMS spectra and the cross-linking intensities (eIF2/TC) are given for every cross-link. Cross-linked amino acid residues are highlighted in red, modified amino acids residues are labelled with * (C → carbamidomethylation, M → oxidation). Cross-links that have been identified only in eIF2 are labelled with “eIF2”. Cross-links highlighted in grey could not be assigned unambiguously.

| **Prot. 1** | **Prot. 2** | **Sequence 1** | **Sequence 2** | **Res. 1** | **Res. 2** | **# Spectra** | **eIF2/TC** |
| --- | --- | --- | --- | --- | --- | --- | --- |
| alpha | alpha | SIQ**K**LIR | VD**K**EK | 61 | 78 | 3 | 0.27 |
|  |  | VG**K**NDVAVVLR | SIQ**K**LIR | 67 | 61 | 21 | 4.27 |
|  |  | VG**K**NDVAVVLR | VD**K**EK | 67 | 78 | 4 | 1.52 |
|  |  | VG**K**NDVAVVLR | GYIDLS**K**R | 67 | 87 | 5 | 4.77 |
|  |  | GYIDLS**K**R | SIQ**K**LIR | 87 | 61 | 15 | 8.06 |
|  |  | GYIDLS**K**R | VD**K**EK | 87 | 78 | 1 | 0.18 |
|  |  | VSSEDII**K**C*EEK | YQ**K**SK | 97 | 104 | 1 | 0.22 |
|  |  | S**K**TVHSILR | LTPQAV**K**IR | 106 | 183 | 1 | 4.74 |
|  |  | YC*AE**K**FQIPLEELYK | NYIS**K**R | 118 | 175 | 1 | 2.71 |
|  |  | V**K**LVAAPLYVLTTQALDK | LTPQAV**K**IR | 221 | 183 | 7 | 2.62 |
|  |  | LVAAPLYVLTTQALD**K**QK | YQ**K**SK | 237 | 104 | 1 | 0.11 |
|  |  | LVAAPLYVLTTQALD**K**QK | NYIS**K**R | 237 | 175 | 3 | 0.52 |
|  |  | Q**K**GIEQLESAIEK | NYIS**K**R | 239 | 175 | 2 | 5.44 |
|  |  | VD**K**E**K**GYIDLSK |  | 78/80 |  | 7 | 0.91 |
|  |  | VSSEDII**K**C*EE**K**YQK |  | 97/101 |  | 1 | 1.12 |
|  |  | YQ**K**S**K**TVHSILR |  | 104/106 |  | 4 | 1.11 |
|  |  | DVLDEL**K**NYIS**K**R |  | 170/175 |  | 2 | 0.81 |
|  |  | LVAAPLYVLTTQALD**K**Q**K**GIEQLESAIEK |  | 237/239 |  | 1 | 0.17 |
|  |  | Q**K**GIEQLESAIE**K**ITEVITK |  | 239/250 |  | 1 | 0.22 |
| alpha | beta | SIQ**K**LIR | DG**K**K | 61 | 170 | 1 | eIF2 |
|  |  | SIQ**K**LIR | LVI**K**GK | 61 | 214 | 3 | 32.96 |
|  |  | SIQ**K**LIR | TGFQATVG**K**R | 61 | 281 | 3 | 132.01 |
|  |  | VD**K**EKGYIDLSK | TGFQATVG**K**R | 78 | 281 | 5 | 4.65 |
| alpha | beta | VD**K**EK | TGFQATVG**K**R | 78 | 281 | 1 | 5.44 |
| alpha | gamma | SIQ**K**LIR | **K**R | 61 | 66 | 6 | 100.33 |
|  |  | SIQ**K**LIR | F**K**DELER | 61 | 129 | 16 | 14.80 |
|  |  | SIQ**K**LIR | NITI**K**LGYANAK | 61 | 139 | 4 | 6.02 |
|  |  | SIQ**K**LIR | SFDVN**K**PGAEIEDLK | 61 | 325 | 5 | 4.56 |
|  |  | VG**K**NDVAVVLR | SFDVN**K**PGAEIEDLK | 67 | 325 | 2 | 2.37 |
|  |  | S**K**TVHSILR | SFDVN**K**PGAEIEDLK | 106 | 325 | 1 | 5.39 |
| beta | beta | **K**TIFSNIQDIAEK | LVI**K**GK | 171 | 214 | 10 | 6.03 |
|  |  | G**K**FQSK | DG**K**K | 216 | 170 | 1 | 5.44 |
|  |  | TGFQATVG**K**R | DG**K**K | 281 | 170 | 1 | eIF2 |
|  |  | TGFQATVG**K**R | LVI**K**GK | 281 | 214 | 8 | 15.59 |
|  |  | **K**S**K**SVSADAEAEK |  | 54/56 |  | 1 | 2.99 |
|  |  | EPTDDIAEALGELSL**KK**K |  | 82/83 |  | 1 | 6.56 |
|  |  | **K**T**K**DSSVDAFEK |  | 87/89 |  | 12 | 12.46 |
|  |  | DSSVDAFE**K**ELA**K**AGLDNVDAESK |  | 98/102 |  | 1 | 0.52 |
|  |  | DG**KK**TIFSNIQDIAEK |  | 170/171 |  | 19 | 6.38 |
|  |  | LVI**K**G**K**FQSK |  | 214/216 |  | 22 | 101.98 |
|  |  | G**K**FQS**K**QMENVLR |  | 216/220 |  | 13 | 7.95 |
| beta | gamma | S**K**SVSADAEAEKEPTDDIAEALGELSLK | **K**R | 56 | 66 | 3 | 1.38 |
|  |  | SVSADAEAEKEPTDDIAEALGELSL**K**K | **K**R | 82 | 66 | 1 | 2.63 |
|  |  | SGP**K**FR | F**K**DELER | 157 | 129 | 3 | 26.74 |
|  |  | SGP**K**FR | GTIADGAPIVPISAQL**K**YNIDAVNEFIVK | 157 | 288 | 1 | eIF2 |
|  |  | YILEYVTC**K**TCK | F**K**DELER | 237 | 129 | 1 | eIF2 |
|  |  | SINTEL**K**R | F**K**DELER | 247 | 129 | 1 | 10.52 |
|  |  | SINTEL**K**R | NITI**K**LGYANAK | 247 | 139 | 1 | 9.09 |
|  |  | SINTEL**K**R | LGYANA**K**IYK | 247 | 146 | 1 | 1.46 |
|  |  | SVSSI**K**TGFQATVGK | F**K**DELER | 272 | 129 | 2 | 4.62 |
|  |  | TGFQATVG**K**R | F**K**DELER | 281 | 129 | 3 | 9.42 |
|  |  |  |  |  |  |  |  |
| gamma | gamma | **K**VAFTGLEEDGETEEEKR | **K**R | 48 | 66 | 5 | 1.60 |
|  |  | VAFTGLEEDGETEEE**K**R | **K**R | 64 | 66 | 18 | 0.16 |
|  |  | VAFTGLEEDGETEEE**K**R | SF**K**SDKEISPK | 64 | 168 | 3 | 2.85 |
|  |  | F**K**DELER | SF**K**SDK | 129 | 165 | 3 | 64.61 |
|  |  | NITI**K**LGYANAK | F**K**DELER | 139 | 129 | 14 | 7.99 |
|  |  | NITI**K**LGYANAK | IE**K**HWR | 139 | 507 | 1 | 8.99 |
|  |  | SF**K**SDK | **K**R | 165 | 66 | 3 | 26.17 |
|  |  | SF**K**SDKEISPK | **K**R | 165 | 66 | 8 | 35.80 |
|  |  | SF**K**SDKEISPK | F**K**DELER | 165 | 129 | 2 | 4.63 |
|  |  | YNIDAVNEFIV**K**TIPVPPR | Y**K**LVR | 300 | 184 | 3 | 5.26 |
|  |  | SFDVN**K**PGAEIEDLK | F**K**DELER | 325 | 129 | 3 | 3.99 |
|  |  | LGDEIEIRPGIVT**K**DDK | SF**K**SDK | 362 | 165 | 1 | 0.54 |
|  |  | LLGV**K**TDGQK | QA**K**VR | 444 | 452 | 4 | 7.47 |
|  |  | RLLGV**K**TDGQK | QA**K**VR | 444 | 452 | 1 | 4.95 |
|  |  | LLGV**K**TDGQK | RIE**K**HWR | 444 | 507 | 2 | 4.71 |
|  |  | **K**LEPNEVLMVNIGSTATGAR | QA**K**VR | 455 | 452 | 2 | 2.04 |
|  |  | **K**LEPNEVLM*VNIGSTATGAR | VVAV**K**ADM*AR | 455 | 479 | 8 | 1.58 |
|  |  | VVAV**K**ADMAR | LIGWATI**K**K | 479 | 518 | 26 | 6.02 |
|  |  | VVAV**K**ADMAR | **K**GTTLEPIA | 479 | 519 | 9 | 7.35 |
|  |  | NITI**K**LGYANA**K**IYK |  | 139/146 |  | 9 | 2.59 |
|  |  | SF**K**SD**K**EISPK |  | 165/168 |  | 17 | 1.23 |
|  |  | EESALEHQ**K**SIL**K**FIR |  | 264/268 |  | 23 | 5.66 |
|  |  | SFDVN**K**PGAEIEDL**K**GGVAGGSILNGVFK |  | 325/334 |  | 1 | 2.00 |
|  |  | LGDEIEIRPGIVT**K**DD**K**GK |  | 362/365 |  | 11 | 2.24 |
|  |  | G**K**IQC***K**PIFSNIVSLFAEQNDLK |  | 367/371 |  | 2 | 0.48 |
|  |  | LLGV**K**TDGQ**K**QAK |  | 444/449 |  | 13 | 27.20 |
|  |  | TDGQ**K**QA**K**VR |  | 449/452 |  | 32 | 32.49 |
|  |  | LIGWATI**KK**GTTLEPIA |  | 518/519 |  | 3 | 8.93 |

Table S7: PTMs listed in Uniprot, PHOSIDA and PTMfunc databases. The residue numbers and the type of PTM (p, phosphorylation; ac, acetylation) are given. PTMs listed in the databases or identified in this study are indicated by ×.

| **Protein** | **PTM** | **Uniprot** | **PHOSIDA** | **PTMfunc** | **this study** |
| --- | --- | --- | --- | --- | --- |
| **eIF2α** | *p*S52 | × |  |  |  |
|  | *p*Y127 |  |  | × |  |
|  | *p*S292 | × | × | × | × |
|  | *p*S294 | × | × | × | × |
|  | *p*S301 |  | × | × | × |
| **eIF2β** | *p*S2 |  | × | × |  |
|  | *p*S40 | × | × | × | × |
|  | *p*S57 |  |  | × |  |
|  | *p*T69 | × |  | × |  |
|  | *p*S80 | × |  | × |  |
|  | *p*T88 |  |  | × |  |
|  | *p*S91 |  |  | × | × |
|  | *p*S92 | × |  | × | × |
|  | *ac*K102 |  |  | × |  |
|  | *p*S112 | × | × | × | × |
|  | *p*T116 | × | × | × |  |
|  | *p*S118 | × |  | × |  |
|  | *p*S121 |  |  | × |  |
|  | *p*S251 |  |  | × |  |
|  | *p*S292 |  | × |  |  |
| **eIF2γ** | *p*T60 | × |  | × | × |
|  | *p*S258 | × |  | × | × |
|  | *p*S312 |  |  | × | × |
|  | *p*T400 |  |  | × |  |
| **eIF2Bα** | *ac*S2 (N-term) | × |  |  | × |
|  | *p*T291 | × |  |  |  |
| **eIF2Bβ** | *p*Y299 |  |  | × |  |
|  | *p*S302 |  |  | × |  |
|  | *p*Y305 |  |  | × |  |
| **eIF2Bγ** | *p*S294 |  | × | × |  |
|  | *p*S296 | × |  | × | × |
|  | *p*S300 | × |  | × |  |
|  | *p*T306 | × |  | × |  |
|  | *p*T316 |  |  | × |  |
| **eIF2Bδ** | *ac*S2 (N-term) | × |  |  |  |
|  | *p*S15 |  |  | × |  |
|  | *p*S106 | × |  |  |  |
|  | *p*S121 | × |  |  |  |
| **eIF2Bε** | *p*S435 |  |  | × | × |
|  | *p*Y445 |  |  | × |  |
|  | *p*S465 |  |  | × |  |
| **eIF2Bε** | *p*Y476 |  |  | × |  |
|  | *p*S478 | × | × | × |  |
|  | *p*S481 | × | × | × |  |
|  | *p*S486 |  |  | × |  |
|  | *p*S507 | × | × | × |  |
|  | *p*S510 |  | × | × |  |
|  | *p*S512 |  | × | × |  |
|  | *p*S513 |  |  | × |  |
|  | *p*T515 |  | × | × |  |
|  | *p*T523 |  |  | × |  |
|  | *p*S525 | × |  | × | × |
|  | *p*S528 |  |  | × |  |
|  | *p*T531 |  |  | × |  |
|  | *p*S538 | × | × | × | × |
|  | *p*S707 | × |  | × |  |
|  | *p*S708 |  | × | × |  |
|  | *p*S709 |  | × | × |  |
